# Special Section Guest Editorial: JBO Special Section on Hyperspectral Imaging

**DOI:** 10.1117/1.JBO.30.2.023501

**Published:** 2025-03-04

**Authors:** Baowei Fei, Jeeseong Hwang, Matija Milanič

**Affiliations:** a University of Texas at Dallas, Richardson, Texas, United States; b UT Southwestern Medical Center, Richardson, Texas, United States; c National Institute of Standards and Technology, Boulder, Colorado, United States; d University of Ljubljana, Ljubljana, Slovenia

## Abstract

The editors introduce a Journal of Biomedical Optics (JBO) special section on Hyperspectral Imaging. The 2-part special section features a number of important research and review papers on new hyperspectral imaging and detection devices and associated technologies, for application in the areas of translational research and clinical studies.

Hyperspectral imaging (HSI) technologies initially have been developed in the field of remote sensing^1^ and have increasingly been applied for a broad range of biomedical applications.^2^ The key analysis goal of hyperspectral data cubes is to determine whether a targeted spectral signature involving disease or anomaly is present or not in the data and to spatially and spectrally resolve the target features. To this end, a variety of analysis algorithms have been demonstrated. With the advancement of AI technologies, especially deep-learning methods, the spectral–spatial analysis of the information-rich HSI data provides promising results that are not easily accessible with conventional machine-learning methods. This JBO special section, published in two parts (Part 1 and Part 2), features a number of important research and review papers on new hyperspectral imaging and detection devices and associated technologies, for application in the areas of translational research and clinical studies.

HSI technologies highlighted in this JBO special section:

(i) **HSI Devices/Systems**. Hyperspectral dark-field microscopy was developed for breast tumor margin detection.^3^ A high-speed hyperspectral laparoscopic imaging system was developed for minimally invasive surgeries.^4^ A transportable HSI system enables rapid biochemical mapping of fresh surgical biopsies.^5^ Push-broom and snapshot HSI cameras were compared for *in vivo* brain tissue analysis.^6^ A multispectral laser speckle contrast imaging system was developed for assessing blood perfusion at multiple depths, utilizing multiple laser wavelengths and advanced correlation techniques to improve depth localization.^7^
(ii) **Machine Learning and AI Tools**. Machine learning and near-infrared spectroscopy data were used for real-time inference of brain tissue composition.^8^ A random forest (RF) algorithm was used to analyze collagen’s architectural changes and associated tumor using mid-infrared spectral imaging and second harmonic generation microscopy.^9^ An artificial neural network was used with HSI to estimate tissue composition and quantify hemoglobin, melanin, and scattering from reflectance spectra.^10^ A vision transformer with HSI data was used for thyroid carcinoma detection in histological slides.^11^ A supervised technique based on the spectral angle mapper algorithm and an unsupervised technique based on the K-means algorithm were applied to classify various tissue types including carcinoma subtypes on hyperspectral dark-field microscopy.^3^
(iii) **Simulation and Analytic Tools**. Using Monte Carlo simulations, a digital simulator was developed to optimize HSI development for intraoperative brain mapping.^12^ Based on the Beer–Lambert law, a molecular composition analysis method was developed to enable real-time inference of biochemical changes of brain tissue.^8^
(iv) **Phantom Designs**. Tissue-mimicking phantoms with the targeted spectral signatures validated by other measurements in parallel and by rigorous physics-based model may be instrumental to establish the measurement ground truth on macroscopically observed optical properties by an HSI device.^13^ Tissue phantoms were designed to study the effects of microstructures on optical properties and light scattering with HSI.^13^ This study also demonstrates the use of phantom microstructure knowledge for absolute measurements of phantom constitution without prior calibration and the definition of the key metrics and associated ground truths of the measurement results is essential for performance evaluation of the imaging devices.

Clinical translation of the HSI devices requires confirmation of their measurement proficiency to identify the tissue’s spectral signatures involving disease and anomaly. This JBO special section highlights multiple applications of HSI:

(i) **Cancer Detection**. Hyperspectral dark-field microscopy was developed to classify tissue types and detect carcinoma in breast lumpectomy samples.^3^ In this study, HSI successfully differentiates carcinoma subtypes in lumpectomy samples. In another study, HSI and transformer networks were explored to enhance the detection of thyroid cancer in histological slides, demonstrating the HSI approach outperforms the conventional RGB-based method.^11^ HSI and spectral renormalization techniques were used to improves tumor microenvironment analysis.^14^ In this study, improved intravital monitoring of dorsal skinfold window chamber models is demonstrated. Multimodal mid-infrared imaging and second harmonic generation microscopy are developed to study collagen in clinical tissues.^9^
(ii) **Surgery Guidance and Monitoring**. A multiconfiguration high-speed hyperspectral laparoscopic imaging system was developed for minimally invasive surgeries with a tissue-classification capability.^4^ A transportable hyperspectral imaging system was designed for real-time biochemical analysis of fresh tissue biopsies.^5^ Near-infrared diffuse spectroscopy and analytic tools were developed to infer molecular composition change of brain tissue, potentially enabling intra-operative monitoring.^8^ A digital simulator was developed to optimizes hyperspectral system development for intraoperative functional brain mapping.^12^ The special issue also includes a review paper on clinical applications of HSI in brain surgery, including tumor classification and perfusion mapping.^15^
(iii) **Tissue Optics and Physiology**. HSI, along with scanning electron microscopy, was used to investigate how tissue-mimicking phantom microstructure affects optical behavior.^13^ HSI and analytic tools were developed to estimate tissue composition, hemoglobin, melanin, and scattering.^10^ Short-wave infrared HSI was also tested to detect biological tissue components like collagen and lipids.^16^ Hyperspectral confocal microscopy was used to quantify gametocytogenesis in malaria-infected red blood cells.^17^ Quantitative measurement of hemoglobin and hemozoin changes provides biomarkers for malaria gametocyte development, aiding antimalarial drug research.^17^
(iv) **Biometric Application**. HSI was explored as a biometric tool for individual identification based on palm vein patterns.^18^

The 16 papers of this JBO special section demonstrate how HSI and AI can improve cancer detection, tissue classification, and surgical guidance. Optical phantom design and tissue modeling will continue to be an active research area to advance HSI development. The special section highlights the importance of advanced HSI techniques in neurosurgical applications where tumor margin assessment, blood oxygenation mapping, and perfusion imaging can play an important role in improving surgical outcome and patient care. Emerging applications include infectious disease research and drug development. With the development of new HSI cameras, sensing technologies, and computational AI tools, hyperspectral imaging will continue to advance biomedical research and clinical care.
